# MicroRNAs in doxorubicin-induced cardiotoxicity: The DNA damage response

**DOI:** 10.3389/fphar.2022.1055911

**Published:** 2022-11-21

**Authors:** Ippei Kawano, Michaela Adamcova

**Affiliations:** Department of Physiology, Faculty of Medicine in Hradec Kralove, Charles University in Prague, Hradec Kralove, Czechia

**Keywords:** microRNA, doxorubicin, cardiotoxicity, genotoxic stress, p53

## Abstract

Doxorubicin (DOX) is a chemotherapeutic drug widely used for cancer treatment, but its use is limited by cardiotoxicity. Although free radicals from redox cycling and free cellular iron have been predominant as the suggested primary pathogenic mechanism, novel evidence has pointed to topoisomerase II inhibition and resultant genotoxic stress as the more fundamental mechanism. Recently, a growing list of microRNAs (miRNAs) has been implicated in DOX-induced cardiotoxicity (DIC). This review summarizes miRNAs reported in the recent literature in the context of DIC. A particular focus is given to miRNAs that regulate cellular responses downstream to DOX-induced DNA damage, especially p53 activation, pro-survival signaling pathway inhibition (e.g., AMPK, AKT, GATA-4, and sirtuin pathways), mitochondrial dysfunction, and ferroptosis. Since these pathways are potential targets for cardioprotection against DOX, an understanding of how miRNAs participate is necessary for developing future therapies.

## Introduction

Doxorubicin (DOX) is an anthracycline (ANT) antibiotic that is widely used as a chemotherapeutic agent against a variety of malignancies, namely breast cancer, prostate cancer, small cell lung carcinoma, Hodgkin’s lymphoma, and others. Despite the high efficacy, its use is significantly limited by its chronic cardiotoxicity. DOX-induced cardiotoxicity (DIC) risk increases as the cumulative dose exceeds 400–700 mg/m^2^ for adults and 300 mg/m^2^ for children (Renu et al., 2018). Patients with DIC could show symptoms ranging from subclinical left ventricular (LV) dysfunction to congestive heart failure (CHF) and death (Bansal et al., 2019). DOX-induced CHF could present acutely (weeks after the onset of treatment) or chronically (decades later), with most cases seen in the first year of therapy (Lipshultz et al., 2008; Cardinale et al., 2015). As DIC is irreversible, dose monitoring and early detection are critical to minimize morbidity and mortality. LV function can be monitored with speckle tracking echocardiography (STE) combined with serum biomarkers such as cardiac troponins and natriuretic peptides to predict DIC risk (Kang et al., 2013; Pudil et al., 2020).

Despite many decades of investigation, the mechanism of DIC has not been completely understood (Mitry and Edwards, 2016). Free radical reactive oxygen species (ROS) generation due to one-electron redox cycling by the quinone moiety of DOX and increased free cellular iron are the most favored hypotheses in the literature (Horenstein et al., 2000; Minotti et al., 2004; Šimůnek et al., 2009). However, other mechanisms have also been suggested, such as DNA damage due to topoisomerase II inhibition and double-stranded breaks (DSBs) (Pommier et al., 2010; Marinello et al., 2018), mitochondrial dysfunction (Štěrba et al., 2011; Osataphan et al., 2020; Wallace et al., 2020; Huang et al., 2022), altered calcium homeostasis (Wallace, 2007; Shinlapawittayatorn et al., 2022), and others. Recently, the ROS hypothesis has been questioned as the primary pathogenic mechanism (Pointon et al., 2010; Zhang et al., 2012; Ghigo et al., 2016; Kalyanaraman, 2020). Instead, inhibition of topoisomerase IIβ (Top2B) in cardiomyocytes and the downstream DNA damage response seems to be the predominant mechanism for mitochondrial dysfunction and ROS generation leading to DIC (Zhang et al., 2012; Vejpongsa and Yeh, 2014). Dexrazoxane, the only drug used in clinics to prevent DIC, has long been thought to achieve its effect through iron chelation (Štěrba et al., 2013). However, more recent evidence points to its interaction with Top2B, not its iron-chelating function, as the primary mechanism for its cardioprotective effect (Kaiserová et al., 2006; Lyu et al., 2007; Deng et al., 2014; Kollárová-Brázdová et al., 2020; Jirkovská et al., 2021; Jirkovský et al., 2021).

Although the understanding that connects DNA damage and DIC is incomplete, certain pathways have been implicated to play a role. Upon DNA damage such as DSBs, cells activate a group of serine/threonine kinases sensitive to DSBs, known as ataxia telangiectasia mutated (ATM), ATM- and RAD3-related (ATR), and DNA-dependent protein kinase (DNA-PK). ATM/ATR can stabilize p53, the master regulator of cell fate after DNA damage (Jackson and Bartek, 2009). In DOX-treated cardiomyocytes, both ATM and p53 are activated (L’Ecuyer et al., 2006; Yoshida et al., 2009; Piegari et al., 2013). Heterozygous p53 loss attenuated DOX-induced cardiomyocyte apoptosis *in vivo*, suggesting p53 activation by DOX contributes to DIC (Yoshida et al., 2009). Meanwhile, DNA-PK is required for the non-homologous end joining (NHEJ) of DSBs. DNA-PK can also activate the pro-survival AKT *via* the mammalian target of rapamycin (mTOR) complex 2 (mTORC2) (Dragoi et al., 2005; Bozulic et al., 2008; Liu et al., 2022). This DNA-PK-AKT axis activation has also been reported in DOX-treated cardiomyocytes (Gratia et al., 2012). While AKT activation may be beneficial for cardiomyocyte survival, in the context of DIC, it is later followed by AKT inhibition (Lee et al., 2006; Das et al., 2011; Sahu et al., 2019). Initial transient AKT activation may rather exacerbate cardiomyocyte energy deficiency and death in DIC by inhibiting AMP-activated protein kinase (AMPK) signaling (Gratia et al., 2012). In addition, the DNA-PK-AKT axis can stabilize p53 following DNA damage, independent of ATM/ATR (Boehme et al., 2008). In summary, DNA damage response in DOX-treated cardiomyocytes likely converges on p53 and AKT/AMPK signaling pathways that, in turn, regulate cell death. One of the major downstream targets of these pathways is the mitochondrion. Specifically, DOX-induced DNA damage and p53 activation may trigger mitochondrial membrane potential loss (L’Ecuyer et al., 2006; Sardão et al., 2009), mitochondrial fragmentation (Samant et al., 2014; Tang et al., 2017; Catanzaro et al., 2019), and impaired mitophagy (Hoshino et al., 2013; Li et al., 2022) in cardiomyocytes, potentiating cell death.

In recent years, microRNA (miRNA) has garnered attention as a novel therapeutic target for DIC (Holmgren et al., 2016; Ruggeri et al., 2018; Skála et al., 2019; Pereira et al., 2020). In the last few years, several experimental and clinical studies have described changes in miRNAs induced by the cardiotoxic effect of ANT with very controversial results (Ruggeri et al., 2018; Pereira et al., 2020; Chen et al., 2021). However, the role miRNAs play in the molecular pathways responsible for DIC is not wholly understood.

This paper aims to give an integrated overview of the impact of miRNAs on the early pathogenetic mechanism of DIC development. Particular attention is paid to the role of the DNA damage response, as it triggers various cellular changes. When the DNA damage exceeds the extent of cellular repair, cells commit themselves to regulated cell death (RCD), including apoptosis and ferroptosis, likely contributing to cardiac functional compromise (Christidi and Brunham, 2021). As a part of RCD, pro-survival signals (e.g., AMPK, AKT, GATA-4, and the sirtuin pathways) are inhibited, and mitochondria become fragmented and accumulate iron within their matrix. Multiple molecular pathways of this process are regulated by miRNAs, raising the possibility of attenuating DOX-induced RCD through targeting miRNAs.

## miRNAs involved in p53 regulation

One of the primary pathways that leads to DOX-induced apoptosis is the oxidative DNA damage-ATM-p53 pathway (Kurz et al., 2004; Yoshida et al., 2009). DNA damage such as DSB activates ATM. One of the most critical downstream targets of ATM is p53, which is activated into a form with improved DNA binding affinity and rapidly accumulates inside of the cell to induce cell cycle arrest, DNA repair, and apoptosis (Lakin and Jackson, 1999; Cheng and Chen, 2010). By causing DSBs and increased ROS generation, DOX can activate this ATM-p53 pathway of DNA damage response (Kurz et al., 2004; Yoshida et al., 2009). p53 was also identified as the critical transcriptome regulator of DIC in a study using transcriptomic profiling, with p53-induced upregulation in death receptors potentially playing a role in increased cardiomyocyte apoptosis (McSweeney et al., 2019). However, the role p53 plays in DIC has also been reported to be cardioprotective through its impact on mitochondrial DNA (mtDNA) rather than apoptosis-inducing activity. Loss of p53 function led to the exacerbation of DIC that can be rescued by recovering mtDNA health (Li et al., 2019b).

The ATM-p53 axis induces the **miR-34** family. Even without DNA damage, a stagnant pool of mature miR-34 is present, quickly activated upon DNA damage by ATM/Clp1-dependent 5′-phosphorylation and Ago2-loading (Salzman et al., 2016). Later, p53 also initiates the transcription of miR-34 (Chang et al., 2007; He et al., 2007; Raver-Shapira et al., 2007; Hermeking, 2012). In humans, three members of miR-34 exist, namely, miR-34a from chromosome 1p36 and miR-36b/c from chromosome 11q23. Among these three, miR-34a has been particularly implicated in the p53 response, as it directly targets *TP53* and its inhibitors *MDM4, YY1, MTA2, HDAC1,* and *SIRT1* (Yamakuchi et al., 2008; Kaller et al., 2011; Navarro and Lieberman, 2015). This double-negative regulation of p53 by miR-34a effectively serves as a positive feedback loop. Recently, a mathematical bifurcation analysis has proposed an exciting function for such a circuit regulating p53 dynamics (Gao et al., 2020), producing limit cycle oscillations needed for biochemical oscillators (Novák and Tyson, 2008; Gao et al., 2020). Therefore, miR-34a can trigger p53 fluctuation *via* its action on sirtuin 1 (SIRT1) mRNA. The amplitude of this activated p53 oscillation is determined by the intensity of DNA damage coded by the activated ATM level and the power of SIRT1 inhibition by miR-34a. A moderate increase in the intensity produces a sustained oscillation of p53 that activates genes involved in cell cycle arrest and DNA repair. However, a significant increase in intensity leads to a prolonged high-level steady state that triggers pro-apoptotic genes (Gao et al., 2020). This agrees with the current experimental data describing how p53 dynamics differentially regulates cell decision-making, between survival (cell cycle arrest, DNA repair) and death (apoptosis), likely through the modulation of its binding affinity to target genes. Pro-apoptotic genes have a lower affinity for p53 than pro-arrest genes (Purvis et al., 2012; Wu et al., 2017). Notably, p53 is not solely regulated by miR-34a to generate an oscillatory pattern, as miR-34 loss did not impair p53 function (Concepcion et al., 2012). Other mechanisms have also been proposed to produce p53 pulses, such as the well-studied p53-mouse double minute 2 homolog (MDM2) feedback loop (Lev Bar-Or et al., 2000; Mihalas et al., 2000) and p53-wild-type p53-induced phosphatase 1 (Wip1)-ATM/checkpoint kinase 2 (Chk2) negative feedback loop (Fiscella et al., 1997; Fujimoto et al., 2006; Shreeram et al., 2006; Boehme et al., 2008). Interestingly, the p53 pulse regulator Wip1 is targeted by miR-16, whose expression is under the regulation of p53 (Zhang et al., 2010; Lezina et al., 2013). This p53-miR16-Wip1 positive feedback loop may also impact cell fate decision (Issler and Mombach, 2017).

Besides the modulation of p53, miR-34a also directly inhibits G1/S transition by targeting *c-Myc, n-Myc, CCND1, E2F, CDK4, CDK6,* and *MET* and induces apoptosis through anti-apoptotic *Bcl-2* and *SIRT1* (Bommer et al., 2007; Yamakuchi et al., 2008; Kaller et al., 2011; Chen and Hu, 2012). Therefore, in addition to regulating cellular p53 dynamics, miR-34a directly impacts cell fate and regulation to aid in p53 function.

Unsurprisingly, after DOX exposure, miR-34a levels increase in rat cardiomyocytes, which is reversed by dexrazoxane (Zhu et al., 2017). In a rat model, antimiR-34a suppressed cardiomyocyte apoptosis *via* upregulating Bcl-2 and SIRT1 and reduced DIC (Piegari et al., 2016, 2020). SIRT1 prevents cardiac apoptosis by inhibiting the pro-apoptotic p66shc, increasing mitochondrial ROS generation (Zhu et al., 2017). Therefore, SIRT1 inhibition by miR-34a can exacerbate DOX-induced cardiac oxidative stress *via* the action of p66shc. Taken together, the miR-34 family, mainly miR-34a, is induced in cardiomyocytes upon DOX-induced DNA damage *via* the ATM-p53 axis. It acts synergistically with p53 to induce cardiac cell cycle arrest and apoptosis, contributing to DIC.

**MiR-23a** and **miR-128** are also associated with p53 in DOX-induced cardiomyocyte apoptosis (Adlakha and Saini, 2013; Li et al., 2015). MiR-23a is upregulated by DOX treatment and exposure to hydrogen peroxide, and it promotes apoptosis by binding to p53 (Li et al., 2015). Upon binding, the complex associates with the miR-128 promoter region, increasing miR-128 expression (Li et al., 2015). An increased miR-128 level was reported in DOX-treated hearts (Zhang et al., 2021b). miR-128 promotes apoptosis by inhibiting SIRT1, resulting in increased p53 acetylation and activity (Adlakha and Saini, 2013; Li et al., 2015). Additionally, miR-128 also contributes to DIC by targeting peroxisome proliferator-activated receptor γ (PPAR-γ), a protective agent against cardiomyocyte oxidative stress and apoptosis (Ren et al., 2009; Zhang et al., 2021b).

Another p53 target that seems to play a role in DIC is **miR-22**. Upon DOX exposure, miR-22 is activated to target the cell cycle regulator p21 (Tsuchiya et al., 2011), which is also activated by p53 during stress conditions. It induces cell cycle arrest by inhibiting various cyclin-dependent kinases, eventually leading to cell senescence rather than apoptosis. However, under high stress, p53 induces the transcription of both miR-22 and p21, thereby suppressing p21 action and favoring apoptosis rather than senescence (Tsuchiya et al., 2011). DOX also induces miR-22 upregulation in murine cardiomyocytes (Xu et al., 2020). miR-22 inhibition has recently been reported to counteract DIC *via* directly targeting SIRT1 (Xu et al., 2020; Wang J et al., 2021). SIRT1 deacetylates the peroxisome proliferator-activated receptor gamma coactivator-1α (PGC-1α), a vital regulator of mitochondrial mass and function (Tang, 2016). It has been reported that when miR-22 is knocked out, SIRT1 suppression is removed, and the expression of PGC-1α and other mitochondrial biogenesis or mitophagy regulators are enhanced, alleviating mitochondrial dysfunction in DIC (Wang R et al., 2021).

In addition to the miRNAs mentioned above, p53 activates other miRNAs, such as miR-200, miR-15/16 family, miR-192/194/215 family, miR-145, and miR-107, although whether they play a role in DIC remains to be largely elusive (Hermeking, 2012). Another form of non-coding RNA, known as large intergenic non-coding RNA-p21 (lincRNA-p21), is also activated by p53 (Huarte et al., 2010). LincRNA-p21 might compete with mRNA for miRNA binding at the miRNA binding sites (MREs), functioning as competing endogenous RNAs (ceRNAs) (Amirinejad et al., 2020). For example, lincRNA-p21 can bind to the miR-181 family, miR-17-5p, miR-1277-5p, and other miRNAs to regulate their levels (Amirinejad et al., 2020). These downstream miRNA targets of p53 might also be altered by DOX treatment in cardiomyocytes.

Lastly, p53 can be therapeutically targeted by **miR-146a**, which is upregulated by DOX treatment (Horie et al., 2010; Pan et al., 2019). miR-146a directly inhibits TATA-binding protein-associated factor 9b (TAF9b), a p53 coactivator and stabilizer (Pan et al., 2019). Reduced p53 stability improves autophagy and reduces apoptosis in DOX-treated cardiomyocytes (Pan et al., 2019). MiR-146a also inhibits cardiomyocyte death by targeting cyclophilin D, which can otherwise trigger mitochondrial permeability transition pore (mPTP) opening and necrosis (Su et al., 2021). Although this mechanism of miR-146a action is not studied in DIC, since cyclophilin D does seem to participate in DIC, the connection is speculated (Dhingra et al., 2020). On the other hand, miR-146a has also been reported to exacerbate acute DIC, especially in patients simultaneously receiving trastuzumab (an anti-ErbB2 monoclonal antibody) through targeting ErbB4 (Horie et al., 2010). This is due to ErbB4 participating in the neuregulin-1 (NRG-1)-ErbB2/ErbB4-phosphoinositide 3-kinase (PI3K)-AKT-mTOR axis that is protective against DIC (Fukazawa et al., 2003; Bian et al., 2009).

## miRNAs involved in the inhibition of pro-survival signaling pathways

### AMPK pathway

How DOX treatment affects cardiac AMPK activity has been controversial. DOX treatment was reported to show activation, no difference, and inhibition of AMPK and autophagy (Koleini and Kardami, 2017; Christidi and Brunham, 2021). One potential hypothesis explaining conflicting observations might be that DOX initially activates AMPK and thus autophagy but later inhibits them. Nevertheless, it is generally accepted that DOX-induced AMPK inhibition plays a role in DIC, and therefore AMPK activation can be cardioprotective against DOX (Gratia et al., 2012; Wang et al., 2012; Timm and Tyler, 2020; Russo et al., 2021). Dysregulated AMPK activity and autophagy can lead to the accumulation of undegraded autophagosomes/autolysosomes, further potentiating cardiomyocyte deaths (Christidi and Brunham, 2021).

The mechanism connecting DOX treatment and AMPK modulation remains elusive, but oxidative and genotoxic stress are likely essential players. In response to DNA damage, DNA-PK can transiently activate the pro-survival AKT pathway, which can at least partially inhibit AMPK (Lee et al., 2006; Gratia et al., 2012). DOX-induced AMPK inhibition may further exacerbate genotoxic stress and cardiomyocyte apoptosis by p53 accumulation and reduced SIRT1 activity due to reduced NAD^+^/NADH ratio (Wang et al., 2012). Through AMPK inhibition, DOX may also attenuate mitochondrial biogenesis and oxidative metabolism *via* PGC-1α, reduce autophagy/mitophagy *via* ULK1, and increase fibrosis *via* TGF-β signaling, i.e., all factors that could contribute to DIC (Timm and Tyler, 2020). AMPK inhibition can at least partially explain metabolic alterations in DOX-treated cardiomyocytes, namely reduced fatty acid oxidation and oxidative phosphorylation, accompanied by persistent glycolysis, potentially culminating in energetic failure (Russo et al., 2021).

**miR-451** might be involved in DOX-induced AMPK inhibition, potentially contributing to DIC (Li et al., 2019a). In DOX-treated mouse cardiomyocytes, the level of miR-451 was significantly enhanced, and the inhibition of miR-451 suppressed cardiomyocyte death and functional compromise (Li et al., 2019a). miR-451 attenuates AMPK signaling, and AMPK inhibition abolishes benefits obtained by miR-451 (Li et al., 2019a). These results indicate that DOX-induced miR-451 upregulation contributes to AMPK inhibition and DIC. Mechanistically, miR-451 might inhibit AMPK signaling by targeting MO25, a component of the upstream kinase of AMPK (Chen et al., 2012).

Another miRNA significantly upregulated by DOX that may alter AMPK signaling is **miR-25**. Recently, it was reported that miR-25 could exacerbate DOX-induced cardiac cell apoptosis and ROS production *via* targeting phosphatase and tensin homolog deleted on chromosome 10 (PTEN) (Li et al., 2020d). PTEN is a negative regulator of AKT signaling, and its loss in the heart leads to impaired AMPK signaling (Roe et al., 2015). Therefore, DOX-induced miR-25 upregulation might attenuate AMPK signaling *via* targeting PTEN. However, as mentioned in the next section, PTEN inhibition and AKT activation have also been reported to be cardioprotective against DIC (Yu et al., 2020; Meng and Xu, 2022). Further testing is required to determine whether the harmful effect of miR-25 depends on the alteration of AMPK or AKT pathways.

### AKT pathway

DOX treatment can initially (2–8 h after treatment) transiently activate and later inhibit the pro-survival PI3K/AKT pathway (Lee et al., 2006; Das et al., 2011; Sahu et al., 2019). There is abundant evidence that suggests upregulating AKT signaling can ameliorate cardiomyocyte apoptosis in DIC (Taniyama and Walsh, 2002; Das et al., 2011; Maruyama et al., 2011; Guo et al., 2013; Cao et al., 2014; Sahu et al., 2019; Zhang et al., 2019; Li et al., 2020a; Zhang et al., 2020; Liao et al., 2022). AKT inhibition can be achieved by p53, which can transcriptionally activate PTEN, the AKT inhibitor (Feng et al., 2007). p53 has also been reported to inhibit mTOR signaling, a primary downstream target of AKT, which could lead to the loss of myocardial mass in DOX-treated hearts (Zhu et al., 2009). Interestingly, DOX-induced AKT inhibition can also be mediated by two miRNAs upregulated by DOX, **miR-143** and **miR-375** (Li et al., 2020b; Liu et al., 2020b). miR-143 upregulation exacerbates oxidative stress and cardiomyocyte apoptosis by inhibiting AKT (Li et al., 2020c). miR-375 upregulation was similarly associated with oxidative stress and cardiomyocyte apoptosis by directly targeting 3-phosphoinositide-dependent protein kinase 1 (PDK1), an AKT activator (Liu et al., 2020b). Inhibition of miR-143 or miR-375 attenuates cardiomyocyte apoptosis in an AKT-dependent fashion (Liu et al., 2020b; Li et al., 2020c).

Meanwhile, some miRNAs have been reported as AKT activators and may counteract DIC. These are, for example, **miR-495-3p, miR-17-5p,** and **miR-21** (Tong et al., 2015; Yu et al., 2020; Meng and Xu, 2022). MiR-495-3p expression is downregulated in DOX-treated hearts, and it targets PTEN to activate AKT signaling (Meng and Xu, 2022). Unlike miR-25, this PTEN inhibition attenuated DOX-induced oxidative stress and DIC. Similarly, miR-17-5p also targets PTEN (Yu et al., 2020). MiR-17-5p may partially be responsible for the cardioprotective effect of dexrazoxane against DOX-induced apoptosis, as miR-17-5p is upregulated by dexrazoxane treatment (Yu et al., 2020). Meanwhile, the expression of miR-21 is increased in cardiomyocytes after DOX treatment. It targets PTEN and B cell translocation gene 2 (BTG2), another negative regulator of the AKT pathway (Yang et al., 2014; Tong et al., 2015).

### GATA-4 pathway

DOX treatment inhibits GATA-4 activity, another critical survival factor that resists DIC (Kim et al., 2003; Kobayashi et al., 2010; Lenčová-Popelová et al., 2014). GATA-4 enhances anti-apoptotic Bcl2 expression, leading to reduced apoptosis and suppressing deleterious DOX-induced autophagy. (Aries et al., 2004; Kobayashi et al., 2010). GATA-4 also plays a role in cellular senescence, activated by ATM and Rad3-related (ATR) upon DNA damage (Kang et al., 2015). This can indicate that GATA-4 action might lead to cardiac senescence and functional impairment. Whether GATA-4 activation is a viable therapeutic strategy remains elusive.

DOX-induced GATA-4 suppression can be mediated by p53, which suppresses CBF/NF-Y binding to the CCAAT box of the promotor region of *GATA-4* (Park et al., 2011). It could also be given by **miR-208a** upregulation, directly targeting GATA-4 (Tony et al., 2015). Hence, miR-208a silencing counteracts such DOX-induced functional compromise (Tony et al., 2015). However, contradicting reports exist regarding miR-208a expression after DOX treatment. Vacchi-Suzzi et al. described downregulation in rat hearts rather than upregulation (Vacchi-Suzzi et al., 2012). Meanwhile, **miR-199a-3p** is another miRNA that directly targets GATA-4, but its level was attenuated in cardiomyocytes by DOX treatment rather than increased (Xia et al., 2021). miR-199a-3p attenuated cardiomyocytes’ senescence and promoted cell proliferation, indicating a potential therapeutic effect in DIC (Xia et al., 2021).

### Sirtuin pathways

Sirtuins are a family of highly conserved NAD^+^-dependent deacetylases and ADP-ribosyltransferases that play an essential role in metabolic regulation and DNA damage repair. There are currently seven members in this family, denoted SIRT1-7, localized in the cytoplasm (SIRT1, 2) or mitochondria (SIRT3, 4, 5) or the nucleus (SIRT1, 2, 6, 7) (Matsushima and Sadoshima, 2015; Mei et al., 2016). Sirtuins could serve a pro-survival role in cardiomyocytes, and at least the involvement of SIRT1, 2, 3, and 6 have been implicated in counteracting DIC (Christidi and Brunham, 2021; Li et al., 2021).

SIRT1 expression is downregulated by DOX exposure in neonatal rat cardiomyocytes, a change associated with increased oxidative stress and apoptosis (Ruan et al., 2015). SIRT1 overexpression or pharmacological activation attenuates DIC through various downstream targets. Specifically, pro-apoptotic p53, as well as p38^MAPK^ and p66shc, are inhibited by SIRT1, while the cardioprotective AMPK is activated by SIRT1 *via* liver kinase B1 (LKB1) and sestrin 2 action (Zhang et al., 2011; Ruan et al., 2015; Wang et al., 2017, 2022; Wu et al., 2019). In addition to the AMPK pathway, SIRT1 activates cardioprotective PGC-1α and nuclear factor erythroid 2-related factor 2 (NRF2). It inhibits NOD-like receptor family pyrin domain-containing protein 3 (NLRP3) inflammasome and nuclear factor kappa-light-chain-enhancer of activated B cells (NF-κB) (Wang(a) et al., 2021). PGC-1α increases mitochondrial biogenesis, while NRF2 transcriptionally activates antioxidant enzymes, thereby contributing to the attenuation of oxidative stress and the alleviation of DIC (Li et al., 2014b; Guo et al., 2014; Zhang et al., 2021a). Meanwhile, NLRP3 inflammasome induces pyroptosis, exacerbating DIC (Meng et al., 2019; Sun et al., 2020).

DOX may inhibit this pro-survival SIRT1 pathway downstream to DNA damage. For example, p53 may inhibit SIRT1 *via* activating hypermethylation in cancer 1 (HIC1) (Wales et al., 1995; Chen et al., 2005). DNA damage-induced poly(ADP-ribose) polymerases (PARPs) activation can deplete NAD^+^, which compromises SIRT1 function (Pillai et al., 2005; Khadka et al., 2018). In addition to these pathways, at least a part of the mechanism involves miRNAs activated by p53 to target SIRT1, namely **miR**-**34a, miR-22,** and **miR-128** (Adlakha and Saini, 2013; Zhu et al., 2017; Xu et al., 2020; Wang J et al., 2021). As previously mentioned, DNA damage by p53 can activate these miRNAs, which regulate p53 activity *via* SIRT1, or target other apoptosis genes. Interestingly, genotoxic stress has been reported to induce ATM-dependent activation of SIRT1, which, by deacetylating histones H1 and H4, promotes the recruitment of DNA repair machinery (Mei et al., 2016). Therefore, SIRT1 inhibition in the DOX-treated hearts may impair DNA repair and potentiate the cell towards apoptotic death.

SIRT2 might also serve a cardioprotective function *via* attenuating oxidative stress by deacetylation/activation of forkhead box O3a (FOXO3a), which upregulates manganese superoxide dismutase (MnSOD) (Matsushima and Sadoshima, 2015). As reported by Zhao et al., the SIRT2 expression is attenuated by DOX treatment and Nrf2 by the action of **miR-140-5p** in cardiomyocytes, thereby leading to increased oxidative stress (Zhao et al., 2018).

SIRT3 suppresses Bcl-2-like 19 kDa-interacting protein 3 (Bnip3), a critical contributor to DIC, by promoting mitophagy and apoptosis (Dhingra et al., 2014; Saito and Sadoshima, 2015; Du et al., 2017). Although no studies report miRNAs targeting SIRT3 in the context of DIC, to our knowledge, **miR-195** may be a candidate. MiR-195 is upregulated in human cardiomyocyte cell lines by DOX treatment, and it might promote cardiomyocyte apoptosis targeting Bcl-2 and SIRT3 (Zhang et al., 2018; Chen et al., 2019a).

SIRT6 attenuates DIC by upregulating endogenous antioxidant levels and attenuating apoptosis (Wu et al., 2021). The latter is achieved by SIRT6 interaction with p53, which leads to the repression of p53 transcription and binding to the Fas/FasL promotor region to reduce apoptotic signaling (Wu et al., 2021). SIRT6 also enhances GATA-4 chromatin binding capacity *via* TIP60 recruitment, counteracting DOX-induced apoptosis (Peng et al., 2020). SIRT6 is targeted by **miR-330-5p**, upregulated in DOX-treated cardiomyocytes (Han et al., 2020). MiR-330-5p exacerbates DIC by inhibiting SIRT6 and the anti-apoptotic survivin and sarcoplasmic reticulum Ca^2+^-ATPase 2a (SERCA2a) (Han et al., 2020). The miR-330-5p sponge circular RNA ITCH (CircITCH) prevents DIC (Han et al., 2020).

## miRNAs involved in DOX-induced mitochondrial fission and mitophagy

DIC is also caused by altered mitochondrial dynamics (Osataphan et al., 2020). Specifically, DOX treatment leads to dynamin-related protein 1 (Drp1)-dependent mitochondrial fission, resulting in enhanced apoptosis propensity (Catanzaro et al., 2019). Indeed, when mitochondrial fission is inhibited through Drp1 knockdown or by the mitochondrial division inhibitor (mdivi-1), it attenuates cardiac cell death and DOX-induced functional compromise (Gharanei et al., 2013; Catanzaro et al., 2019).

Mitochondrial fission can be induced by p53, which can transcriptionally activate Drp1 (Li et al., 2010). This process is counteracted by **miR-30** and **miR-499-5p,** two cardioprotective miRNAs downregulated in the heart after DOX treatment (Li et al., 2010; Roca-Alonso et al., 2015; Wan et al., 2019). miR-30 family downregulates p53 expression, reducing Drp1-mediated mitochondrial fission and apoptosis (Li et al., 2010). Of note, miR-30 can also counteract DIC by targeting β1-and β2-adrenoceptors (β1AR, β2AR) and Giα-2 of the *β*-Adrenergic signaling pathway, as well as the pro-apoptotic *BNIP3L/NIX* (Roca-Alonso et al., 2015). Furthermore, miR-30a and miR-30e target Beclin-1 to preserve cardiomyocyte autophagy and avoid DOX-induced apoptosis (Lai et al., 2017; Zhang et al., 2021c). Meanwhile, miR-499-5p directly targets p21 and both *α*- and *β*-Isoforms of the calcineurin subunits, which favor mitochondrial fission by increasing Drp1 dephosphorylation and accumulation in mitochondria (Wang et al., 2010; Wan et al., 2019). Interestingly, miR-499 expression is transcriptionally suppressed by p53 (Wang et al., 2010). To summarize, DOX treatment downregulates miR-30 while also causing DNA damage, leading to p53 activation. p53, in turn, suppresses miR-499-5p as well as transcriptionally activating Drp1, resulting in increased mitochondrial fission and apoptosis.

DOX-induced mitochondrial fragmentation is also mediated by **miR-532-3p, miR-23a,** and **miR-140** (Li et al., 2014a; Wang et al., 2015; Du et al., 2019). miR-532-3p is upregulated by DOX and targets apoptosis repressor with caspase recruitment domain (ARC), a critical negative regulator of mitochondrial fission and apoptosis (Wang et al., 2015). This miR-532-3p-ARC axis of DOX-induced apoptosis was reported to be present only in cardiomyocytes but not in cancer cells. In addition to the pro-apoptotic role *via* binding to p53, DOX-induced miR-23a expression favors mitochondrial fission and cardiomyocyte apoptosis by directly targeting PGC-1α, an inhibitor of Drp1 phosphorylation and activation (Du et al., 2019). Meanwhile, **miR-140**, another miRNA upregulated by DOX in cardiomyocytes, targets and downregulates the pro-fusion protein mitofusin 1 (Mfn1) rather than manipulating Drp1 activity, aiding in mitochondrial fission and cardiomyocyte apoptosis (Li et al., 2014a).

Another aspect of mitochondrial dysfunction in DIC is dysregulated mitophagy. Mitophagy refers to mitochondrial autophagy, or the selective engulfment of damaged mitochondria into autophagosomes for lysosomal degradation (Bravo-San Pedro et al., 2017). Impairing mitophagy leads to the accumulation of damaged mitochondria and compromises cellular bioenergetics. In cardiomyocytes, two significant pathways induce mitophagy: PTEN-induced kinase 1 (PINK1)/Parkin pathway and Bnip3/NIX pathway (Koleini and Kardami, 2017). While Bnip3/NIX-dependent mitophagy’s role in DIC is elusive, contradictory findings have been reported for the PINK1/Parkin pathway in DIC. Some of them described that DIC is due to DOX attenuating Parkin-dependent mitophagy (Hoshino et al., 2013), while others the opposite (Yin et al., 2018; Catanzaro et al., 2019). Hoshino et al. (2013) showed that DOX upregulates cytosolic p53, which directly binds to Parkin and prevents its translocation to mitochondria. They, therefore, suggest that increased mitophagy may be therapeutic for DIC. In contrast, Yin et al. (2018) and Catanzaro et al. (2019) reported DOX activates Parkin-mediated mitophagy, and inhibition of mitophagy by mdivi-1 or Parkin knockdown mitigated DIC. The discrepancies may be due to differences in experimental conditions.

Reports on the role of miRNA in DOX-induced mitophagy are scarce. One recent study showed that **miR-147-y** may counter DOX-induced cardiomyocyte death by augmenting mitophagy (Gao et al., 2022). miR-147-y achieves its effect by targeting Raptor, a subunit of mTOR complex 1 (mTORC1) and a potential negative regulator of mitophagy (Gao et al., 2022). Another recent article found that DOX suppresses mitophagy by reducing **miR-152-3p** expression *via* DNA methyltransferase 1 (DNMT1) (Deng et al., 2022). When DNMT1 is inactive, miR-153-3p enhances mitophagy by targeting E26 transformation specific-1 (ETS1) (Deng et al., 2022). Both studies seem to support the idea that DOX suppresses cardiac mitophagy.

## miRNAs involved in DOX-induced ferroptosis

The close relationship between cardiac iron accumulation and DIC has been widely discussed in the literature. DOX quinone moiety redox cycling and inactivation of iron regulatory proteins (IRP1/2) have been reported as the primary mechanistic connection (Minotti et al., 2001; Xu et al., 2005; Šimůnek et al., 2009; Gammella et al., 2014). More recently, DIC has been mainly linked to mitochondrial iron accumulation (Ichikawa et al., 2014). Mechanistically, the dysfunction of the mitochondrial iron exporter ABCB8 correlates with mitochondrial iron level, cellular ROS, and DIC severity (Ichikawa et al., 2014; Menon and Kim, 2022). Impaired ABCB8 function could be induced by iron overload, resulting in increased DOX accumulation in the mitochondria (Menon and Kim, 2022). Mitochondrial iron exacerbated DIC independently on Top2B.

Iron overload is associated with cardiomyocyte ferroptosis (Sumneang et al., 2020). Ferroptosis is a relatively recently discovered mechanism of RCD, and it involves intracellular iron-dependent accumulation of lipid peroxides and impaired antioxidant activity of glutathione peroxidase 4 (GPx4) (Dixon et al., 2012; Li et al., 2020a). It was recently reported that DOX downregulates GPx4 in addition to forming a complex with iron in the mitochondria to promote lipid peroxidation, culminating in mitochondria-dependent ferroptosis (Tadokoro et al., 2020). Significantly, the inhibition of ferroptosis by ferrostatin-1 combined with the inhibition of apoptosis by zVAD-FMK prevented DOX-induced cardiomyocyte death, while using either one only achieved partial prevention (Tadokoro et al., 2020). Since ferrostatin-1 alone or zVAD-FMK alone both seem to account for a significant fraction of DOX-induced cardiomyocyte death, both ferroptosis and apoptosis appear to be the primary forms of cell death in DIC (Tadokoro et al., 2020).

Ferroptosis could also be potentiated by p53, although the interaction is complex, with some p53 downstream targets favoring and others inhibiting ferroptosis (Liu et al., 2020a; Liu and Gu, 2022). Part of the pro-ferroptosis mechanism concerns p53’s impact on cysteine metabolism. Cysteine is an essential component of glutathione (GSH), the main antioxidant required for GPx4 activity. Therefore, a lack of intracellular cysteine leads to the potentiation of ferroptosis (Badgley et al., 2020; Poltorack and Dixon, 2022). Specifically, p53 represses the expression of *SLC7A11*, a cystine/glutamate transporter, leading to reduced cystine (Jiang et al., 2015). p53 also represses ELAVL1 expression, stabilizing the posttranscriptional level of LINC00336, an endogenous sponge for **miR-6852** (Wang et al., 2019). miR-6852 targets cystathionine-β-synthase (CBS) mRNA, which codes the enzyme converting homocysteine to cystathionine reducing intracellular cysteine. Furthermore, p53 also induces the long noncoding RNA (lncRNA) PVT1, an endogenous sponge for miR-214 (Lu et al., 2020; Olivero et al., 2020)**. miR-214** attenuates ferroptosis *via* directly targeting transferrin receptor 1 (TfR1), PVT1, and p53 (Lu et al., 2020). However, others report that miR-214-3p enhances ferroptosis by targeting activating transcription factor 4 (ATF4) (Bai et al., 2020). AFT4 is a transcriptional activator that counteracts ferroptosis through its downstream targets, namely SLC7A11 and heat shock protein family A member 5 (HSPA5), which is a GPx4 activator (Chen et al., 2017b; Chen et al., 2017a; Chen et al., 2019b). The role of these miRNAs is yet to be reported in the context of DOX-induced ferroptosis, a topic that awaits future studies.

NRF2 might be another target of regulation by miRNAs in DOX-induced cardiomyocyte ferroptosis. NRF2 is a transcriptional activator of antioxidant proteins, and its action counteracts ferroptosis by preventing lipid peroxidation and free iron accumulation (Dodson et al., 2019). In addition to this anti-ferroptotic effect, NRF2 can also ameliorate DIC by maintaining autophagy (Li et al., 2014b). NRF2 action is enhanced by **miR-152**, which directly targets Keap1, the inhibitor of NRF2 (Zhang et al., 2021a). miR-152 ameliorates DIC by attenuating oxidative stress and apoptosis, potentially preventing ferroptosis (Zhang et al., 2021a). Furthermore, miR-152 may suppress ferroptosis by targeting TfR1 (Kindrat et al., 2016). Interestingly, NRF2 also transcriptionally represses miR-214, disinhibiting AFT4 and preventing ferroptosis (Gao et al., 2016).

The miRNA regulation of DOX-induced ferroptosis is a poorly understood topic, and the significance of the miRNAs mentioned above is speculative. One miRNA that has been reported to participate in DOX-induced ferroptosis is **miR-7-5p**. DOX treatment upregulates methyltransferase-like 14 (METTL14), which stabilizes lncRNA KCNQ1OT1, a miR-7-5p sponge (Zhuang et al., 2021). Since miR-7-5p directly targets TfR1, KCNQ1OT1 action disinhibits TfR1, contributing to iron accumulation and ferroptosis. Indeed, miR-7-5p mimic prevented DOX-induced iron accumulation and lipid ROS generation (Zhuang et al., 2021).

## MiRNA-based therapeutics for cancer

Increasing evidence shows that altered miRNA expression profiles and unique miRNA signaling pathways are present in different types of cancers. Some miRNAs can function as oncogenes (known as oncomiRs) or tumor suppressors (TS-miRs) during tumor development and progression (Garzon et al., 2009; Lee and Dutta, 2009; Ventura and Jacks, 2009; Wang and Wu, 2012; di Leva et al., 2014). OncomiRs such as the miR-17–92 cluster, miR-21, miR-106a, miR-155, miR-372, and miR-373 target and inhibit various tumor suppressor genes, thereby enhancing cancer survival and proliferation (Zhang et al., 2007; Wang and Wu, 2012). It is not surprising that some of them, such as miR-17-5p and miR-21, also favor cell survival and mitigate DIC in the heart (Tong et al., 2015; Yu et al., 2020). On the other hand, TS-miRs such as miR-15a/16-1, Let-7, and miR-34a counter cell proliferation and favor apoptosis (Zhang et al., 2007; Lee and Dutta, 2009; Ventura and Jacks, 2009; Wang and Wu, 2012). Some of them, such as miR-34a, may also exacerbate cardiomyocyte apoptosis (Piegari et al., 2016, 2020). In the recent decade, anti-oncomiRs and TS-miR mimics have emerged as novel therapeutic strategies to normalize the gene regulatory network and signaling pathways and reverse the phenotype in cancer cells (Forterre et al., 2020). For example, the TS-miR miR-34a is considered to be a potential tumor marker and a promising cancer therapeutic candidate (Kalfert et al., 2020). Although the phase 1 study of MRX34, a liposomal miR-34a mimic, in patients with advanced solid tumors was closed early, miRNA-based gene therapy provides an attractive anti-tumor approach for integrated cancer therapy (Hong et al., 2020). If these therapeutics were to be combined with DOX, specific anti-cancer miRNAs might simultaneously exacerbate DIC. On the other hand, miRNAs that may mitigate DIC can also enhance cancer cell survival. Therefore, it is crucial to develop a highly specific delivery system that minimizes unintentional miRNA effects.

## Conclusion

**FIGURE 1:**
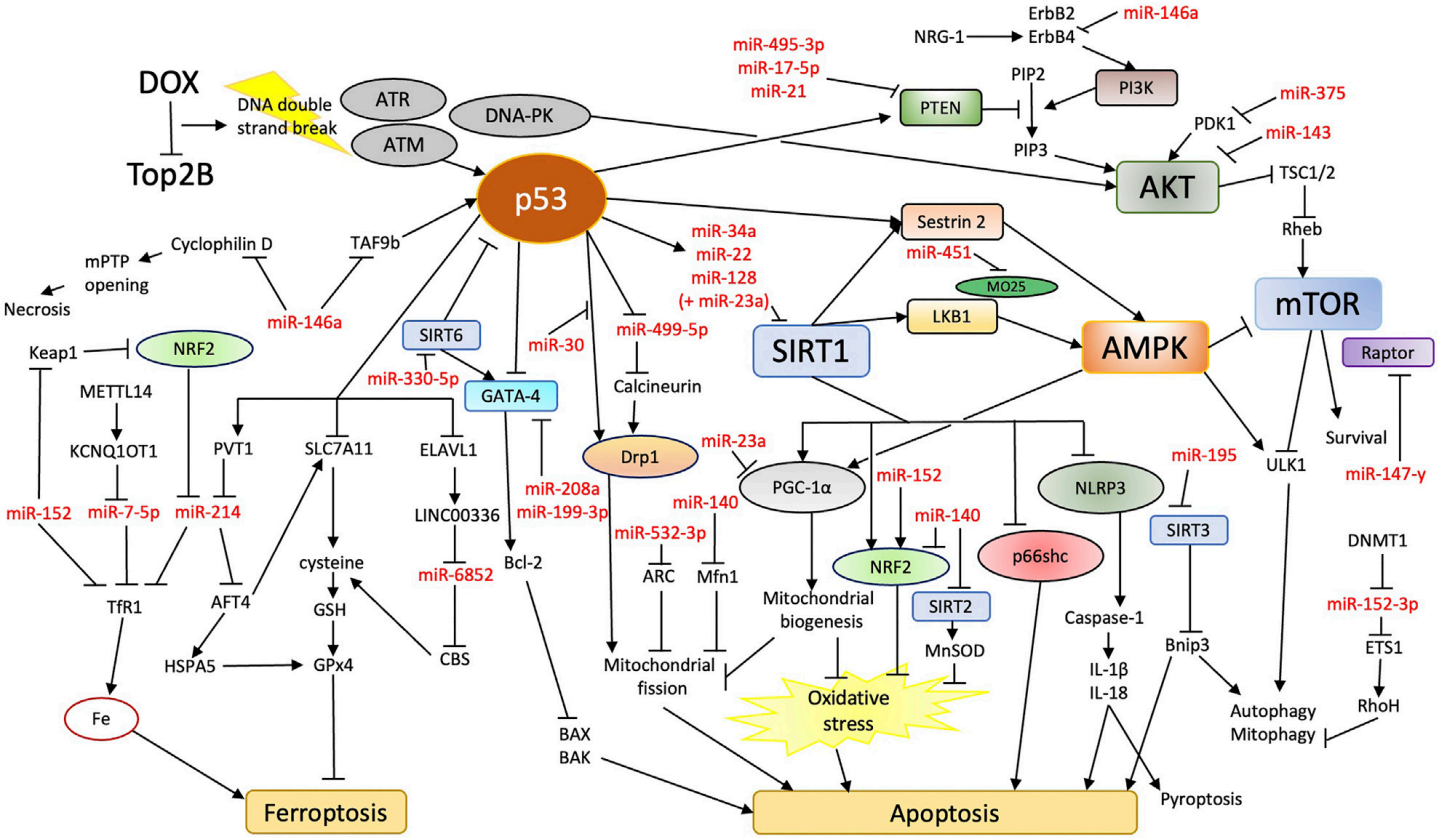
Overview of potential miRNA involvement in DIC. DOX treatment increases apoptosis and ferroptosis in cardiomyocytes. Rather than oxidative stress, DOX inhibition of Top2B and subsequent DNA damage response may be the primary contributor to cardiomyocyte death. miRNAs may aid in the regulation of molecular pathways that connect DNA damage response and apoptosis/ferroptosis.

DNA damage response is increasingly recognized as the essential pathogenic mechanism of DIC, which should be reflected in our current understanding of the role of miRNAs. Increasing evidence points to the hypothesis that excess genotoxic stress in the heart due to DOX treatment may alter miRNA expression to promote RCDs, mainly apoptosis and ferroptosis. We have reviewed evidence suggesting miRNAs may impact RCDs *via* directly modulating p53 dynamics and various pro-survival signaling pathways (Figure 1; Table 1). Counteracting RCD-promoting miRNA changes, therefore, may prevent DIC. Certain aspects of this hypothesis, such as the mutual regulation between p53 and miRNAs in DIC, still require further experimental confirmation.

**TABLE 1:** List of miRNAs potentially involved in DIC.

miRNA	After DOX treatment	Organism/cell	Targets	References
miR-34a	Upregulated	Rat cardiomyocytes	SIRT1, Bcl-2, TP53, MDM4, YY1, MTA2, HDAC1, c-Myc, n-Myc, CCND1, E2F, CDK4, CDK6, MET	Bommer et al. (2007), Yamakuchi et al. (2008), Kaller et al. (2011), Chen and Hu (2012), Navarro and Lieberman (2015), Piegari et al. (2016), Piegari et al. (2020)
miR-23a	Upregulated	Rat cardiomyocytes, primary neonatal rat ventricular myocytes	PGC-1α, (Complexes with p53 to increase miR-128 expression)	Li et al. (2015), Du et al. (2019)
miR-128	Upregulated	Mouse cardiomyocytes	SIRT1, PPAR-γ	Ren et al. (2009), Zhang et al. (2021b)
miR-22	Upregulated	Human colon cancer cells	p21	Tsuchiya et al. (2011)
		Mouse cardiomyocytes	SIRT1	Xu et al. (2020), Wang R et al. (2021)
miR-146a	Upregulated	Mouse cardiomyocytes	ErbB4, TAF9b, cyclophilin D	Horie et al. (2010), Pan et al. (2019), Su et al. (2021)
miR-451	Upregulated	Mouse cardiomyocytes	MO25	Chen et al. (2012), Li et al. (2019a)
miR-25	Upregulated	Mouse cardiomyocytes, H9c2 rat cardiomyoblasts	PTEN	Li et al. (2020d)
miR-143	Upregulated	Mouse cardiomyocytes	AKT	Li et al. (2020c)
miR-375	Upregulated	Mouse cardiomyocytes, H9c2 rat cardiomyoblasts, adult murine cardiomyocytes	PDK1	Liu et al. (2020b)
miR-495-3p	Downregulated	Mouse cardiomyocytes	PTEN	Meng and Xu (2022)
miR-17-5p	Downregulated	Mouse cardiomyocytes	PTEN	Yu et al. (2020)
miR-21	Upregulated	Mouse cardiomyocytes, H9c2 rat cardiomyoblasts	PTEN, BTG2	Yang et al. (2014), Tong et al. (2015)
miR-208a	Upregulated	Mouse cardiomyocytes	GATA-4	Tony et al. (2015)
	Downregulated	Rat cardiomyocytes		Vacchi-Suzzi et al. (2012)
miR-199a-3p	Downregulated	Mouse cardiomyocytes, human-induced pluripotent cell-derived cardiomyocytes	GATA-4	Xia et al. (2021)
miR-140-5p	Upregulated	Rat/mouse cardiomyocytes, H9c2 rat cardiomyoblasts	Nrf2, SIRT2	Zhao et al. (2018)
miR-195	Upregulated	AC16 human cardiomyocytes	Bcl-2, SIRT3	Zhang et al. (2018), Chen et al. (2019a)
miR-330-5p	Upregulated	Human-induced pluripotent cell-derived cardiomyocytes	SIRT6, survivin, SERCA2a	Han et al. (2020)
miR-30	Downregulated	Adult rat ventricular cardiomyocytes, H9c2 rat cardiomyoblasts, neonatal rat cardiac cells	β1AR, β2AR, (Downregulates p53)	Li et al. (2010), Roca-Alonso et al. (2015)
miR-30a	Downregulated	Rat cardiomyocytes	Beclin-1	Lai et al. (2017), Zhang et al. (2021c)
miR-30e	Downregulated	Rat cardiomyocytes	Beclin-1	Lai et al. (2017)
miR-499-5p	Downregulated	Mouse cardiomyocytes, H9c2 rat cardiomyoblasts	p21, α-/β-isoforms of calcineurin subunits	Wang et al. (2010), Wan et al. (2019)
miR-532-3p	Upregulated	Neonatal rat and mouse cardiomyocytes	ARC	Wang et al. (2015)
miR-140	Upregulated	Neonatal rat cardiac cells	Mfn1	Li et al. (2014a)
miR-147-y	—	Freshly isolated neonatal pig cardiomyocytes	Raptor	Gao et al. (2022)
miR-152-3p	Downregulated	H9c2 rat cardiomyoblasts, rat heart	ETS1	Deng et al. (2022)
miR-6852	—	A549 and H358 lung cancer cells	CBS	Wang et al. (2019)
miR-214	—	Human and mouse brain	TfR1, PVT1, p53	Lu et al. (2020)
miR-152	Downregulated	Mouse cardiomyocytes	Keap1, TfR1	Kindrat et al. (2016), Zhang et al. (2021a)
miR-7-5p	Inhibited	AC16 human cardiomyocytes, neonatal rat ventricle cardiomyocytes	TfR1	Zhuang et al. (2021)

It is also necessary to highlight the effects of the same miRNA in proliferating cancer cells and non-proliferating cardiomyocytes. Although the role of miRNAs in carcinogenesis is relatively well understood, the data about the DIC are inconsistent. Furthermore, mice and rat models are currently predominant in the literature, so confirming the findings in human patients would be ultimately required for clinical translation.
